# Examining the Importance of Family History in Pediatric Behavioural Referrals

**DOI:** 10.7759/cureus.4790

**Published:** 2019-05-31

**Authors:** Sarah Gander, Sarah A Campbell, Kathryn E Flood, Emma Grace Crowley

**Affiliations:** 1 Pediatrics, Saint John Regional Hospital, Saint John, CAN; 2 Pediatrics Research, Saint John Regional Hospital, Saint John, CAN

**Keywords:** attention deficit hyperactivity disorder, attention, academic, behaviour, referrals, pediatrics, mother-child relationship, social pediatrics, attention deficit hyperactivity disorder, socio-economic status, attention, academic, behaviour

## Abstract

In recent years, there has been a substantial increase in the diagnosis of attention deficit hyperactivity disorder (ADHD) in children. Without appropriate management of symptoms and care, ADHD has been associated with a variety of negative child and adult outcomes. Environmental and familial factors that may contribute to three different pediatric referral types (academic, behavioural, and attentional) associated with ADHD were examined in the current study. In total, data from 477 families who were interviewed as a part of the intake process to a pediatric clinic were included in this study. Data for the current study was extracted from the intake questionnaires and included information on family history of mental health issues, socioeconomic status, and family relationships. The sample included children between the ages of three and 17 and mostly comprised males (*n* = 340). A frequency analysis of the data demonstrated relatively high rates of mental health issues within families (61.4%); almost half of the mothers reported some post-secondary education (46.1%) and most reported having normal relationships with their children (mothers, 78%; fathers, 62.9%). Finally, three stepwise regression analyses were conducted to predict referral type. All three regressions yielded significant models. Fifteen percent of the variability of the academic referral type was predicted by being male, age at the time of referral, mother’s education level, and mother’s learning. The behavioural referral types were predicted by a family history of depression, being male, mother-child relationship, and age at the time of referral; these accounted for 23% of the variance. Attentional referral type was predicted only by mother-child relationship that captured 6% of the variance. Overall, this study describes a population of parents of children with academic, behavioural, and attention-related referrals to pediatrics. Results indicate that mothers have a profound influence on their child’s referral types, something that may transfer into later diagnosis and perhaps prognosis. Clinicians and researchers alike should focus their efforts toward developing integrative service assessment and treatment approaches that include important people in the child’s life. The implementation of Community Social Pediatrics (a streamlined, inclusive approach to care) should be considered in urban centres like this one, where referrals like this are prevalent.

## Introduction

Attention-deficit hyperactivity disorder (ADHD) is an increasingly diagnosed behavioural disorder characterized by many symptoms including an inability to focus, increased distractibility, and forgetfulness [1]. The implications of these symptoms over time have been associated with educational difficulties, relationship issues, and increased engagement in risky behaviours [2]. In addition, comorbid diagnosis of other conditions (i.e., anxiety, depression, substance use, oppositional defiant disorder, and borderline personality disorder) are common with ADHD [3]. It is imperative to identify and manage these behaviours early, as a lack of treatment can lead to a poor prognosis for ADHD symptoms in adulthood. These symptoms also may be indicative of the underlying environmental or familial stressors being encountered by the child that mirror or augment ADHD symptoms, indicating that consultant pediatricians should consider and evaluate additional indicators (i.e., socioeconomic status) that may be contributing to ADHD symptomology [4].

Socioeconomic status (SES) encompasses factors such as income, education, family history, and geographic location, among others [5]. Individuals who report lower SES may have certain barriers accessing appropriate health care versus individuals with higher SES who are equipped with certain tools (e.g., education, transportation, etc.) because of their background. More specifically, children and families from vulnerable populations may not have the skills to confidently navigate the healthcare system even if they recognize a need for support. This is often due to a lack of knowledge in their rights, healthcare systems, and even normal child development [6]. Factors like low SES may be mediating delays in diagnoses for these vulnerable families, resulting in even poorer outcomes for the child.

Although somewhat heritable, ADHD severity may be influenced by an individual’s early environment. Environmental risk factors include maternal pregnancy complications, low child socialization and nutritional deficits [7]. Moreover, parental mental illness and childhood psychiatric conditions (e.g., ADHD) have been positively associated. That is, children of parents with certain mental illnesses (i.e., bipolar disorder, schizophrenia, depression, alcohol- or substance-use disorder) may be more susceptible to the development of disorders like ADHD [8-13]. Recently, a study conducted by López Seco and colleagues suggested that half of the children in their sample with ADHD had at least one parent with a psychiatric history [14]. Taken together, the importance of understanding familial and social health factors may be integral to early detection and appropriately -tailored treatment of disorders like ADHD. By enhancing our knowledge of a child’s familial situation, clinicians may be able to provide more targeted care and implement useful social programs. In addition, by understanding the relations between parental mental illness and child behaviour issues, we could be able to screen and treat both parents and children in tandem, which may lead to better outcomes overall.

The present study was conducted in a medium-sized Atlantic Canadian city with an urban core of approximately 70,000 residents, but providing pediatric services to a catchment of almost 300,000. The child poverty rate in this area is one of the highest in Canada, at 30%, almost double the national average. Additionally, half of all single-family households in NB live in poverty compared to approximately 10% of two-parent households [15]. Thus, research surrounding SES, behavioural disorders, and familial histories are timely given the situation this city is facing.

The current study examined the family mental-health histories and SES of children who have received pediatric referrals for behaviour-related issues. Through a deeper understanding of the familial demographics in this referral base, clinicians may be able to design and implement family-specific interventions, ultimately aiding in decreasing barriers to care. Furthermore, by understanding situational factors like SES that may be predictive of certain referral types early on, clinicians and other community partners may be able to target and treat vulnerable children earlier which could lead to better outcomes and less time without support and guidance.

## Materials and methods

Participants

In total, 477 families were interviewed pre-pediatric visit as a part of the intake process. Interviews were administered by a clinic nurse, with a parent or guardian of the children being referred to the clinic.

Materials

Data for the current study was extracted from comprehensive intake questionnaires developed by local pediatricians (see Appendix 1). The intake interview was administered to patient parents/guardians between the years of 2012 and 2017 and contained questions regarding medical, familial, and neurodevelopmental histories. The primary purpose of these interviews was to provide pediatricians with a comprehensive picture of a child’s history and needs. Therefore, because research was not originally intended, ethical approval for secondary use of data was granted from the Horizon Health Network’s Research Ethics Board. Lastly, statistical analyses were conducted using IBM SPSS statistical software, Version 22.

Procedure

The data were collected via telephone intake interviews of children being referred to pediatricians for behaviour-related referrals. Interview questions explored details pertaining to the child’s medical, academic and family history and other factors. The telephone interview, conducted by a clinical nurse, was done with a primary caregiver in the child’s life, such as a parent, grandparent, custodial guardian. Information from the interviews was input into a database and appropriately coded for analyses. Descriptive statistics were noted for all variables to understand the sample. Finally, three stepwise regressions were conducted to predict referral type (i.e., academic, behavioural, or attention) using SES and other familial information collected in the interviews.

## Results

The current sample consisted of children ranging from three to 17 years old, 340 males (*M*AGE= 7.57) and 137 females (*M*AGE = 7.41). Over half (62.9%) of the children being referred for behavioural, academic, or attention concerns had a family history of mental health issues. Mental illness was further categorized by disorder or illness type (Table 1). In the current sample, depression was the most prevalent followed by substance use disorder. 

**Table 1:** Percentage of family histories of mental health issues by category

Family History of Mental Illness	Percentage of Sample
Mental Health Issues	62.9%
Anxiety	33.8%
Depression	49.4%
ADHD	31.8%
Learning Problems	23.2%
Manic Depression	2.2%
Schizophrenia	10.6%
Bipolar Disorder	18.0%
Substance Use Disorder	44.9%
Anger Management Issues	37.6%

Factors related to SES also were collected, including levels of parental education, current employment, issues with employment and custody status. Almost half (47.8%) of the mothers in the current sample had at least some post-secondary education, while 52.2% of mothers reported having no post-secondary education with 15% having not graduated high school. Similarly, 35.3% of fathers had some post-secondary education, although, 21.6% indicated they had not graduated high school. Furthermore, at the time of intake, 36.2% of mothers and 21% of fathers were unemployed, and 24.4% of mothers and 24.7% of fathers reported issues with maintaining employment. Most of the children were in the custody of both parents (44.5%), although 41.8% were in the custody of their mother only, 4.5% father only, 7.1% shared parental custody, and 0.9% other.

The intake interview also inquired about the perceived relationship between parents and children, while many families reported relatively normal relationships; however, there were some levels of indifference and conflict recorded as well (Table 2). The frequency of family shared meals was relatively high, 78.0% suggesting that they usually shared a meal as a family. Lastly, 24.8% of families reported receiving some type of parental training.

**Table 2:** Percentage of parent-child relationship types in the current sample

	Mother-Child Relationship	Father-Child Relationship
Normal	84.4%	69.7%
Conflicted	14.5%	13.6%
Indifferent	1.1%	16.6%

Finally, three stepwise regressions were conducted entering SES and relationship variables into the regression equation to predict referral type (i.e., academic, behavioural, and attention). Stepwise regressions were performed as exploratory analyses given the retrospective use of the initial data, to provide a base for future prediction equation modelling by eliminating variables that were not predictive of referral type. Significant models were found for all referral types. Four significant models were found for academic referral type, although the overall model accounted for 15% of the variance in academic referrals (*F* (4, 134) = 6.17), *p* <0.0001). That is, being male, age at the time of referral, mothers with lower education levels and mothers who reported prior learning issues were predictive of academic referral type (Table 3).

**Table 3:** Stepwise regression predicting academic referral type **p < 0.01, *p < 0.05

		Academic Referral				
	R		Β	t	F change	p
STEP 1						R^2 ^=.06
Gender	0.26**		0.26	3.18	10.11	0.002
STEP 2						ΔR^2^=.09
Age at Referral	0.31**		0.03	2.05	7.29	0.001
STEP 3						ΔR^2^=.12
Mother’s Education	0.35*		0.06	2.01	6.31	0.033
STEP 4						ΔR^2^=.15
Mother’s Learning	0.39*		0.23	2.27	6.17	

Stepwise analyses also suggested that these factors may be predictive of behavioural referral types. Four significant models emerged, model 4 accounted for 23% of the variability in behavioural referral type. A positive family history of depression, being male, an “abnormal” mother-child relationship, and a younger age at referral were predictive of a behavioural referral (*F* (4, 136) = 10.26), *p *<0. 0001) (Table 4).

**Table 4:** Stepwise regression predicting behavioural referral type **p < 0.01, *p < 0.05

		Behavioural Referral				
	R		Β	t	F change	p
STEP 1						R^2 ^= 0.08
Family history of depression	0.30**		0.29	3.72	13.87	0.000
STEP 2						ΔR^20^= 0.15
Gender	0.39**		-0.27	-3.25	12.71	0.000
STEP 3						ΔR^2 ^= 0.20
Relationship with Mother	0.44**		0.22	2.73	11.37	0.000
STEP 4						ΔR^2 ^= 0.23
Age at Referral	.48**		-0.03	-2.39	10.26	0.000

Only one model emerged to predict attention referral types, that is, mother-child relationship (Table 5). That is, relationship with mother accounted for 6% of the variance in attention type referrals (*F *(1, 137) = 8.78), *p* < 0.004).

**Table 5:** Stepwise regression predicting attention referral types **p < 0.01

		Attention Referral				
	R		Β	t	F	p
						R^2 ^= 0.06
Relationship with Mother	0.25**		-.027	-2.96	8.78	0.004

## Discussion

The number of children who are experiencing issues related to behaviour, attention, academic, and social difficulties is reaching a critical point [16]. When these children are referred to pediatricians and other clinicians, they often encounter long wait times and a fragmented system of services and resources. Service providers are ill-equipped to consider the complex social and family histories of these children and address these challenges in their assessments and care pathways. Through developing a better understanding of the underlying issues that contribute to children’s difficulties, we hope to identify better treatment paths and to more adequately address the full complement of needs that are relevant to children and their families, discussed further below.

Overall, this study described a population of parents of children with academic, behavioural, and attention-related referrals to pediatricians. Being male was predictive of increased academic and behavioural referral types, which is unsurprising given most of the current sample was composed of male referrals and meta-analytic results that have suggested males present more external problems related to disorders like ADHD (i.e., hyperactivity, inattention, and impulsivity) [17]. Younger age also was a significant predictor of behavioural referral type, though it is important to note that despite ages ranging from 3 to 17, 78.8% of the sample was under the age of nine, which may be due to adolescents being referred to other types of clinicians (e.g., psychiatrists, psychologists, social workers, etc.).

In Canada, 50% of individuals report issues with mental health at some point in their lives [18]. Therefore, the fact that 62.9% of families in this study have a family history of mental illness is not necessarily unusual. Future research could include a more in-depth screening to identify the number of children with parental mental health issues, given the current data focused on entire family history. This aligns with previous research suggesting that this population often require services from other allied health professionals [19], suggesting that these cases are complex and require the application of a more coordinated assessment method.

The role of mother-child relationships have long been identified as a key factor in child development and beyond. Studies have demonstrated that nurture plays a key role in many aspects of development later informing adult experiences (e.g., future relationships, health outcomes, and life satisfaction [20-24]). Impaired mother-child relationship was a significant predictor of attention and behaviour-related referrals in the current study. ADHD symptoms have been linked to mother-child relationship before, one study suggested that there was an association with increased ADHD symptoms and hostility in mother-child relationships, controlling for biological factors [24]. Further, one study suggested that mothers with depression may have children diagnosed with ADHD who demonstrate increased behavioural issues over and above mothers of children with ADHD who report lower levels of depressive symptoms [25]. These are just a few of the associations found between mothers and their child’s behavioural and attention-related issues, warranting further investigation. More specifically, future research should note the child's later diagnosis after the referral process in order to make additional inferences about these relations. Finally, these results do not necessarily suggest that the mother-child relationship is more important than the relationship between the father and their child. Conversely, it should be noted that 41.8% of the current sample was in the custody of their mothers only, while only 4.5% were in the custody of their father's only; given these results, direct comparisons cannot be made. 

Although mother-child relationship did not emerge as a significant predictor of academic referral types, it is interesting to note that the mother’s education and learning history did emerge as predictors of academic referrals. This result suggests that a mother’s history may impact a child’s performance over and above other factors, specifically, their father’s history. It is clear that mothers have a significant impact on their children’s referral type and the relation with the child’s eventual or future diagnoses within the current sample should be explored. Perhaps, future research should focus on the importance of parent-child relationships and other familial and environmental factors when examining pediatric referral types and potential correlates, specifically how these are measured.

The current study was limited in that it was conducted using secondary data developed for clinical, and not research, purposes. Further to the point, the use of stepwise regression analyses is not ideal given the increased number of variables entered into the equation to find the model best fit; however, this method may provide future researchers with knowledge to make informed a priori hypotheses and ultimately capture a larger amount of variance in referral types using these (e.g., parent-child relationship, family history of depression, etc.) and other predictors.

Given the current results, another limitation of this retrospective data was the operationalization of certain variables like parent-child relationship. In the current study, parent-child relationship was measured by simply asking if the child’s relationship with their mother or father (from the guardian’s perspective) was “normal, conflicted, or indifferent”, although this is useful for clinical purposes, it would be more valuable to use psychometrically validated measures in the future. For example, measuring a child’s attachment (i.e., bonds formed with certain individuals like caregivers that inform a variety of an individual’s behaviours throughout their lifespan) with their caregiver may better illustrate the influence of parent-child relationships on pediatric referral types [20-23]. Family and child history also may be better captured through a child and even a guardian’s Adverse Childhood Experience (ACE) scores, simultaneously guiding the clinician to alternative treatment pathways [25]. Regardless, this intake interview has proven to be a valuable tool for informing future research and we feel can now be refined further and paired with psychometric tools for optimal results. 

It is beneficial for pediatric clinicians to understand markers of some of the more common conditions that parents and guardians reported (i.e., depression, anger management, substance dependence, and anxiety) but also, how the presence of these conditions in caregivers may be affecting the child’s health and well-being. Further, a guardian suffering from mental illness may make child treatment plans increasingly difficult for caregivers to effectively navigate with their child. By understanding mental health concerns in families, we can introduce family-based solutions to improve compliance.

Increasingly, there are many examples in practice throughout Canada and globally that stress the importance of the psychosocial model and incorporating the needs and strengths of the family in planning for care and treatment. The Community Social Pediatrics (CSP) model stresses the importance of coming together around the child as caregivers to support the child to realize their full potential [26]. This study illustrates the importance of engaging caregivers in the optimization of their own mental health to meet their child’s needs. This includes promoting the importance of caregiver-child relationships to provide a safe and nurturing environment for development. It recognizes there may be an early warning for children at risk for attention and behavioural problems and that early intervention will likely provide better outcomes. 

A CSP approach offers a streamlined pathway to care for children, specifically those from low SES families where communication across services tends to break down, creating further problems including access to timely care and misdiagnosis. CSP models have demonstrated positive results wherein children demonstrate improvements in their academic grades and overall sense of well-being. It is well-documented children facing social disparities, trauma and toxic stress will experience a disproportionate number of negative physical and mental health outcomes across their lifespan [6,26-29].

## Conclusions

Future researchers and clinicians should focus on this model to build a team that would perform a comprehensive assessment of the child and build a care and treatment plan that considers the entire situation of the child. The implications this approach may have on these children may involve not only short-term benefits (i.e., better health, academic success) but a more inclusive picture of the child’s risk factors (e.g., ACEs), allowing professionals to intervene in a timely manner and ultimately decrease the child’s use of the system throughout their lifespan. 

## Appendices

**Table 6:** Nurse intake form

Name:		Date:
Pediatrician:		
DOB (dd/mm/yy)		
Medicare:		
School:		
Grade:		
Teacher:		
Home meds:		
Pharmacy:		Insurance:

	Email	Phone
Mother		
Father		
Stepfather		
Custodial Parent		

SNAP Forms			
Informant			
Brother(s)			
Sister(s)			
Dept of Social Development			
Foster Care			
Referral Source			
By:			
Concerns:			

Resources in place	Name	Dates involved
Family Doctor		
Psychologist		
PsychEd Assessment		
Mental Health Services		
Parent Training		
Addictions Counselling		
Anger Management		
Alternative Therapy		
Early Intervention		
OT		
PT		
SLP		

Pregnancy		
Maternal Age:		
Gestation at Delivery	EGA:	Birth Weight:
Hypertenison		
Diabetes		
Infections		
Cig/EtOH/drugs		
Gestational Problems		
Postnatal complications		
Development		
Gross Motor		
Fine Motor		
Language		
Socialization		
Vision/Hearing		
Dental		
Other		

Past Medical History		
	No	Yes
Past Medical Diagnosis		
Admissions		
Surgery		
Psychiatric		
Allergies		
Immunizations		
Other		

Review of Systems	
Skin (eczema, rash)	
ENT (ear, throat, nose)	
Respiratory (day or night cough)	
Gastrointestinal	
Genitourinary	
Cardiovascular	
Musculoskeletal	
Hematological	
Neurological	
Puberty	
Birthmarks/ Hair Growths	

Daycare/School History	Years attended/ Concerns raised/ Teacher name
Daycare/preschool	
Kindergarten	
Grade 1	
Grade 2	

Function Assessment	
Sleep, Hygiene (Bedtime, waking, sleep habits)	
Toileting	
Eating (family habits, meal times)	
Relationship / c parents	
Regular chores	
Friends	
Gets along well with peers	
Homework	
Learning	
Written output	
Participation	
Academic Grades	
Classroom behaviour	
Adaptability	
Gets along well with elders	
Ability to be independent / organized	
Weekly exercise	
Free-time behaviour	
Extracurricular activities	
Sports involvement	
Afterschool activity	
Plays with pets	
Handles being teased	
Socially appropriate	
Humour	
Sexual behaviour	
Emotional coping	

Does your child have any of the following?	Yes	No
Shortness of breath during exercise (more than other kids their age) and without any other reason like asthma, obesity, not exercising regularly.		
Poor exercise tolerance (when compared to other kids their age)		
Fainting or seizure with exercise or if startled		
Palpitations (skipped heartbeat) with exercise		
Family members (brothers, sisters, cousins, etc.) with any of the following:		
Long QT syndrome		
Wolff Parkinson White syndrome		
Cardiomyopathy		
Heart transplant		
Pulmonary Hypertension		
Unexplained death or death by sudden drowning or car accident		
Sudden infant death syndrome		
Implanted defibrillator		
Have you ever been told your child has:		
High blood pressure on exam		
Murmur heard on exam		
Needed heart surgery		
